# Efficient generation of *FVII* gene knockout mice using CRISPR/Cas9 nuclease and truncated guided RNAs

**DOI:** 10.1038/srep25199

**Published:** 2016-05-03

**Authors:** Liyou An, Yeshu Hu, Shiwei Chang, Xiumei Zhu, Pingping Ling, Fenli Zhang, Jiao Liu, Yanhong Liu, Yexiang Chen, Lan Yang, Giorgio Antonio Presicce, Fuliang Du

**Affiliations:** 1 Jiangsu Key Laboratory for Molecular and Medical Biotechnology, College of Life Sciences, Nanjing Normal University, Nanjing 210046, P R China; 2 Lannuo Biotechnologies Wuxi Inc., Wuxi 214000, P R China; 3 ARSIAL, Rome, Italy; 4 Renova Life, Inc., College Park, Maryland 20742, USA

## Abstract

We investigated the effects of 5′-end truncated CRISPR RNA-guided Cas9 nuclease (tru-RGN, 17/18 nucleotides) on genome editing capability in NIH/3T3 cells, and its efficiencies on generating Factor VII (*FVII*) gene-knockout (KO) mice. In cultured cells, RGNs on-target editing activity had been varied when gRNAs was truncated, higher at Site Two (tF7–2 vs. F7–2, 49.5 vs. 30.1%) while lower in other two sites (Site One, tF7–1 vs.F7–1, 12.1 vs. 23.6%; Site Three, tF7–3 vs.F7–3, 7.7 vs 10.9%) (P < 0.05). Out of 15 predicated off–target sites, tru-RGNs showed significantly decreased frequencies at 5 sites. By microinjecting tru-RGN RNAs into zygotes, *FVII* KO mice were generated with higher efficiency at Site Two (80.1 vs. 35.8%) and Site One (55.0 vs 3.7%) (*P* < 0.05), but not at Site three (39.4 vs 27.8%) (P > 0.05) when compared with standard RGN controls. Knockout *FVII* mice demonstrated a delayed prothrombin time and decreased plasma FVII expression. Our study first demonstrates that truncated gRNAs to 18 complementary nucleotides and Cas9 nucleases, can effectively generate *FVII* gene KO mice with a significantly higher efficiency in a site-dependent manner. In addition, the off-target frequency was much lower in KO mice than in cell lines via RGN expression vector-mediated genome editing.

Clustered, regularly interspaced, short palindromic repeat (CRISPR) RNA-guided nucleases (RGNs) can robustly induce genome editing^1^,^2^,^3^. Repair of RGN-induced double-stranded breaks by nonhomologous end-joining or homology repair, introduces insertion or deletion mutations (indels) or specific sequence alterations^4^. The *Streptococcus pyogenes* Cas9 nuclease (Cas9) cleaves the intervening spacer sequence directed by a single guide RNA (gRNA) with a 20 nucleotides (nt) target complementarity region, at its 5′ end. However, the frequency and magnitude of undesired off-target mutagenesis can be induced at the sites of sequence, similar to the on-target site^2^,^5^.

Several strategies to improve specificity of the Cas9 system have been reported, such as the paired Cas9 nickase approach, in which two gRNAs target adjacent sites on opposite DNA strands, and each recruit a Cas9 nickase that nicks DNA instead of cutting both strands. This method can reduce off-target modifications at sites induced by single gRNA-guided Cas9^6^,^7^,^8^. Nevertheless, off-target mutations are still observed, and an additional gRNA could introduce new potential sites of off-target mutations. Each single gRNA can independently nick DNA at off-target sites, causing unwanted genome-wide mutations. The dimeric CRISPR RNA-guided *Fok*I nuclease (RFN) strategy can reduce off-target mutations to undetectable levels, but it also reduces indel frequencies on targets^9^,^10^,^11^. Paired Cas9 nickase and RFNs need to be designed appropriately and oriented with their paired gRNAs, which may differ throughout the target gene, and present technical challenges in multiplex applications^12^.

Recent reports demonstrated that truncated gRNAs (tru-gRNAs) improved Cas9 nuclease specificity in U2OS.GFP and FT-HEK293 cells, by shortening the gRNAs to 17/18 nt^12^,^13^. They found that 5′-end nucleotides are not required for standard gRNA (std-gRNAs, 20 nt) activity, and compensate for mismatches at unwanted positions along with the gRNA target DNA interface, as shorter gRNAs are more sensitive to mismatches and therefore exhibit higher specificity. This hypothesis is proven in cultured cells, and it is not sure whether tru-RGNs can result in a high efficiency in generating gene knockout (KO) in animal models. Here, we investigated the activity and specificity of tru-RGNs in inducing coagulation factor VII (*FVII*) gene mutations in murine cells and in generating KO mice by zygote RNA microinjection.

## Results

### Tru-RGNs induce genome editing in murine cells

**Figure 1:**
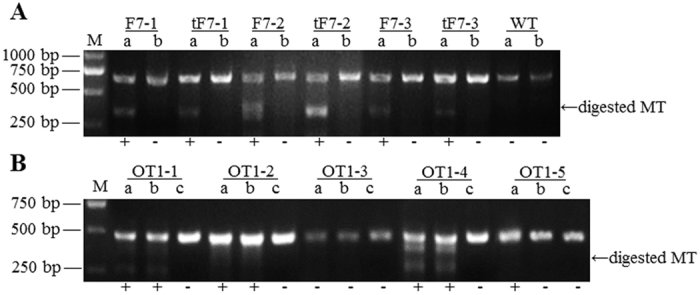
Analysis of RGN-induced genomic mutations in NIH/3T3 cells. (**A**) PCR amplicons of target sites in the *FVII* in cells transfected with RGN expression plasmids (F7–1, F7–2, F7–3, tF7–1, tF7–2, and tF7–3) were subjected to T7EI assays. In each RGN group, T7EI-digested samples (a) and untreated controls (b) were shown. Samples of wild type (WT) cells served as negative controls. (**B**) Off-target cleavage induced by RGNs of F7–1 (a) and tF7–1 (b) at five potential off-target sites (OT1–1, OT1–2, OT1–3, OT1–4, and OT1–5) in transfected cells were detected. Samples from WT cells served as controls (c). M, marker. The fragments of T7EI digested mutant type (MT) were indicated out by arrow. The target and off-target sites were subsequently confirmed by PCR-sequencing.

Tru-RGNs (Site One, tF7–1, 18 nt; Site Two, tF7–2, 18 nt; and Site Three, tF7–3, 17 nt) target three different sites of at the exon 2 of *FVII* gene (Supplementary Fig. S1A,B), and corresponding std-RGNs (F7–1, 43–63; F7–2, 46–66; and F7–3,67–87, all 20 nt, in the exon 2) (Table 1) expression vectors were constructed as controls. Colony efficiency was determined as 0.43–0.53% among three tru-RGN and three std-RGN plasmids (P > 0.05) (Supplementary Fig. S1C). To determine efficiency and specificity of RGN-mediated genome mutations, tru-RGN and std-RGN plasmids were transfected into murine NIH/3T3 cells, and on-target mutations in the *FVII* gene were determined by the T7EI assay (Fig. 1A) and confirmed by sequencing. Genomic mutations were detected in cell population transfected with different RGN plasmids (Table 1). Tru-RGNs of Site Two tF7–2 yielded the highest editing frequencies in all vectors, and significantly higher than std-RGNs of F7–2 (49.5 *vs.* 30.1%). However, both Tru-RGNs of Site One tF7–1 (12.1 *vs.* 23.6%) and Site Three tF7–3 (7.7 *vs.* 10.9%) exhibited reduced editing activities compared to their corresponding std-RGNs, respectively (P < 0.05) (Table 1).

### Tru-RGNs significantly induce genome editing in mice in a site-dependent manner

**Figure 2:**
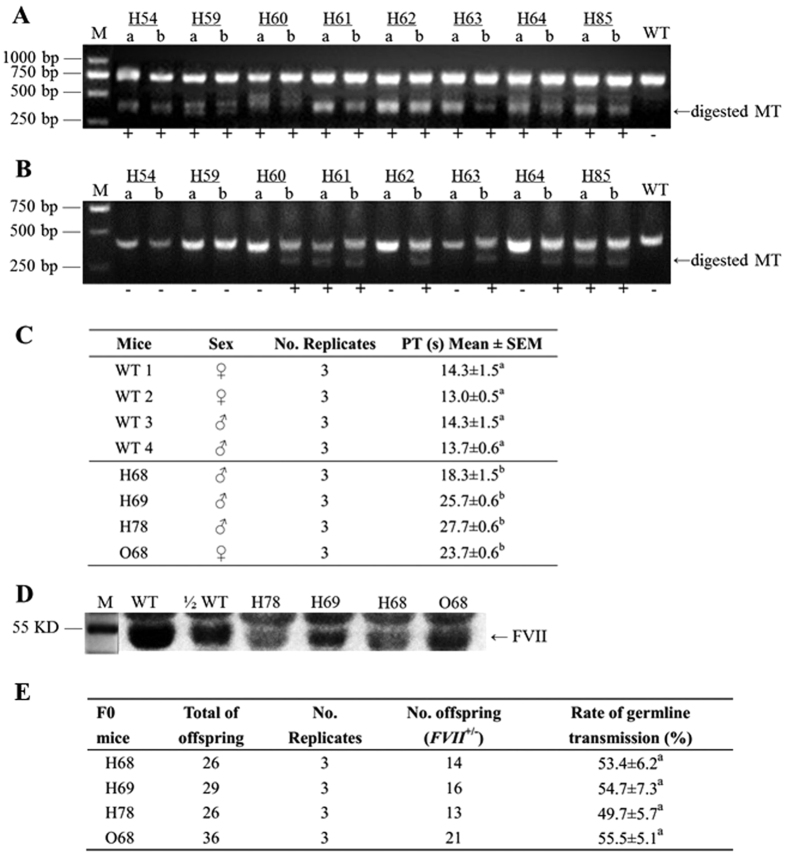
Analysis of genomic mutations in mice. (**A**) PCR amplicons of target sites in murine *FVII* were subjected to T7E I assays. The amplicons from mutant mice (a) and from mutant mice mixed with wild type (WT) amplicons (b, 1:1) were digested to identify biallelic mutations. Amplicons from WT mice served as negative controls (c). (**B**) Amplicons of off-target site (OT3–2) from *FVII* mutant mouse were subjected to T7E I digestion (a). The amplicons were mixed with WT amplicons(1:1) and digested to identify biallelic mutations (b). Amplicons from WT mice were served as negative controls. The target and off-target sites were subsequently confirmed by either PCR-sequencing or TA cloning-sequencing. (**C**) Detection of plasma prothrombin time (PT) in *FVII* mutant mice. a, b values with the different numbers within the same table column were indicative of significant differences (*P* < 0.05). Plasma from four WT mice served as controls. (**D**) Western blot analysis of FVII expression in plasma of mutant mice. Total plasma proteins were separated by SDS-PAGE, and FVII expression was detected by Western blot. The samples H78, H69, H68, and O68 showed a decreased FVII expression to an extent lower than its 1/2 WT level. FVII proteins in Western blot were indicated out by arrow. (**E**) Founder mice (H78, H69, H68, and O68) were breed by mating with wild-type mice, and the rates of germline transmission were recorded. ^a^ values within the same column showed no significant differences (*P* > 0.05).

To determine the editing efficiency of tru-RGNs in generating gene-modified mice, tru-gRNAs and Cas9 mRNA were co-injected into pronuclear stage zygotes to produce *FVII* KO mice. Std-gRNAs (50 ng/μL) and Cas9 mRNA (100 ng/μL) were injected into zygotes as controls. Ninety-eight, 75, and 120 embryos were injected, and 38, 20, and 20 newborns were born from the tru-RGN groups (tF7–1, tF7–2, and tF7–3, respectively). From the std-RGN groups (F7–1, F7–2, and F7–3), 78, 110, and 80 embryos were injected, and 18, 24, and 6 newborns were obtained, respectively (Table 2). Mutagenesis of the newborns was detected by T7EI assays (Fig. 2A) and confirmed by sequencing. In order to distinguish the monoallelic and biallelic mutations, T7EI assays were implemented by mixing equal amount of PCR amplicons over newborns and wild type mice. The results of generating KO mice clearly indicated the site specific and dependent mutations in newborns mediated by tru-RGNs compared to std-RGNs controls. At Site One, the percentage of mice which carried *FVII* indel mutations mediated by tF7–1 tru-RGNs was much higher when compared to std-RGNs F7–1 (55.0 vs. 3.7%, P < 0.05). Similarly at Site Two, tF7–2 tru-RGNs induced the highest percentage of indel mutations (80.1%) in all three target sites, which is significantly higher than its F7–2 std-RGNs controls (35.8%) (P < 0.05). At Site Three, both F7–3 tru-RGNs and F7–3 std-RGNs induced similar percentage of mutations (39.4 vs. 27.8%, P > 0.05). In tru-RGNs groups of tF7–1, tF7–2, and tF7–3 KO mice, 1, 15 and 8 mice contained monoallelic mutations, whereas 18, 0 and 0 mice carried biallelic mutations, respectively. The std-RGNs groups of F7–1, F7–2, and F7–3 resulted in 1, 2 and 2 mice with monoallelic mutations, and 0, 6 and 0 mice with biallelic mutations, respectively (Table 2).

**Figure 3:**
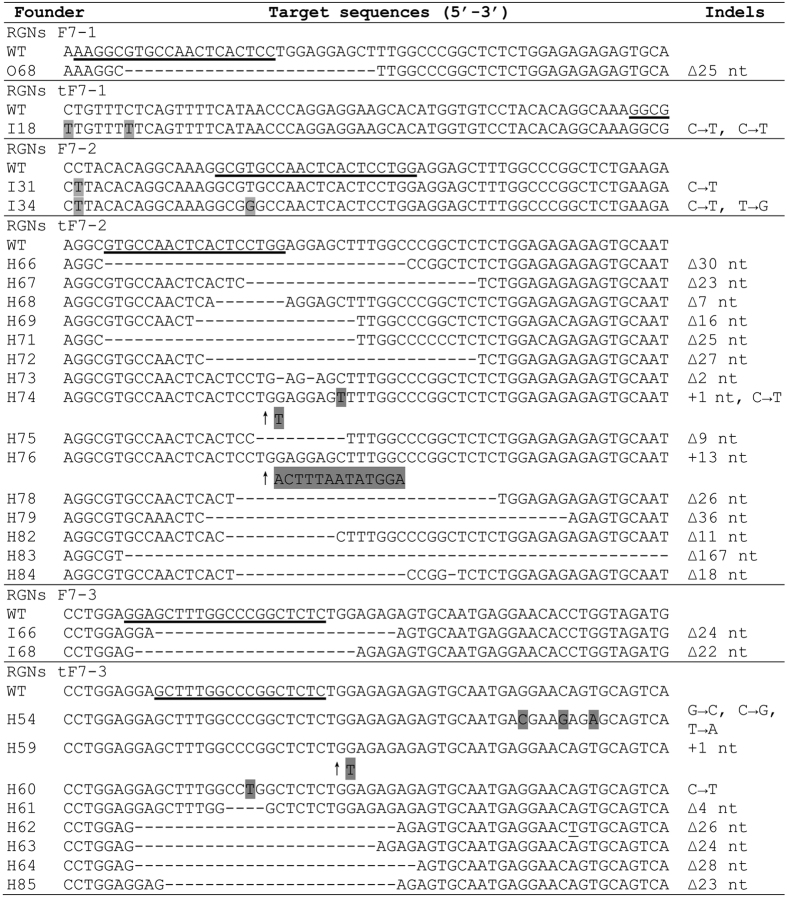
DNA sequences of *FVII* gene mutations induced by RGNs in mice. The guide RNA sequences were labeled as underlines in wildtype. Mutations of nucleotide (nt) transition, transversion, and insertion were marked in grey highlighted, nucleotide deletions were shown as “−”.

We further analyzed and confirmed the modified target sites by DNA sequencing and found that the mutations mainly included deletions, insertions, nucleotide transition and transversion (Fig. 3).

### Off-target mutagenesis induced by tru-RGNs in transfected cells and mutant mice

Potential off-target sites were predicated using the MIT Design Tool. Five off-target sites with the highest homology and affinity to each tru-gRNA at each site were subjected to mutation analysis with T7EI assays. In NIH/3T3 cells, off-target mutation frequencies induced by tru-RGNs at most predicated sites had been decreased in a different degree compared to std-RGNs (Table 3). As a result, at a total of 15 predicated off-target sites, the editing frequencies at 5 sites were decreased significantly (5/15, OT1–5, OT2–1, OT2–5, OT3–4 and OT3–5) (*P* < 0.05) while off-target frequencies decreased but without statistical differences at 4 sites (4/15, OT1–1, OT1–2, OT1–4 and OT3–3) (Fig 1B). There were 5 sites without off-target mutations (5/15, OT1–3, OT2–2, OT2–3, OT2–4 and OT3–1). However, only one off-target mutation at OT3–2 site mediated by tru-RGNs of tF7–3 had a significantly increased frequency when compared to std-RGNs control (22.0 vs. 3.7%, P < 0.05) (Table 3). Six mice generated with tru-RGNs of tF7–3 carried off-target mutations at one predicated site (OT3–2, Fig. 2B; Supplementary Table S2), with its off-target ratio of 29.3% in mutant mice versus newborns (n = 20). In other sites, both tru-RGNs and std-RGNs did not generate off-target mutations in *FVII* KO mice (Table 3).

### Phenotype changes in tru-RGN-mutagenized mice

Mice in tru-RGN group with biallelic mutations died in few days after birth (Table 2), while mice with monoallelic mutations were all alive but suffering deformation in limb in various degree of severity. Similar symptoms of limb deformation and newborn death were observed in std-RGNs groups. It was reported that homozygous *FVII*^*−/−*^ neonates which were generated by homologous recombination, suffered of fatal-abdominal bleeding, and all died within 24 d^30^. In tru-RGN-modified mice (H68, H69, H78 and O68), the plasma phenotype of prothrombin time (PT) was significantly prolonged compared to WT mice (*P* < 0.05) (Fig. 2C). The FVII protein expression in plasma of these four mice was further determined by Western blot, with its level decreased to about 1/2 in wild type plasma in all PT-prolonged KO mice (Fig. 2D). Deduced amino acid sequences of mutant locus from these heterozygous *FVII*^+/−^ mice (H68, H69, H78 and O68), indicated the frame shift of translation at the sites of mutations, and all showed the pre-mature termination of protein translation (Supplementary Table S1). We further mated H68, H69, H78 and O68 with wild-type mice (*FVII*^+/+^). We found that the ratio of heterozygous *FVII*^+/−^ offspring mice was among 49.7–55.5% which followed Mendelian hereditary pattern (Fig. 2E).

## Discussion

Our study has successfully demonstrated, for the first time, that gene KO mice can be generated using tru-RGNs microinjection to fertilized eggs, at the highest efficiency of 80.1%. Fu *et al.*^13^ reported that the truncated guide RNAs significantly improves CRISPR-Cas9 nuclease specificity in human cell lines. It was also reported that Std-RGNs can mediate genome modification in one-celled embryos and generate KO mice^14^,^15^,^16^,^17^. In our study, we found that the ratios of KO mice in newborns using tru-gRNAs, at Site One and Site Two are significantly higher than std-gRNAs, which reveals that tru-gRNAs are more efficient in generating gene KO mice by RNA embryo microinjection.

We have compared the genome editing frequencies of tru-RGNs and std-RGNs in mouse NIH/3T3 cells. Results show that RGNs editing activity varied to a certain extent when 5′-end complementary nucleotides of gRNAs were truncated, with an increase at one target site (Site Two) and a decrease at other sites (Site One and Site Three). These results support previous findings in human cells^13^,^18^. The genome mutation frequencies induced by RGNs are site-dependent, when using both tru-gRNAs and std-gRNAs. In most cases, the ratio of off-target mutations mediated by tru-RGNs in cell lines were decreased, but varied in a certain degree, with a significant decrease at 5 sites. This conforms that magnitudes of sensitivity to mismatches are site-dependent. Fu *et al.*^13^ reported that tru-gRNAs can dramatically decrease undesired mutagenesis at off-target sites in human U2OS.EGFP and FT-HEK293 cells, dependent upon cell lines and targeting sites. They also suggested that lengthening the 5′ end of gRNA reduces on-target editing efficiency, as 5′ nucleotides might not be necessary for full gRNA activity. Thus, shortening the gRNA can potentially decrease off-target effects 5000-fold or more in human cells^13^. A subsequent study further showed that both the number of off-target sites and mutation frequencies are decreased using tru-RGNs as detected by GUIDE-Seq^19^. In contrast, we did not observe such large magnitude of editing improvement in mouse NIH/3T3 at three designated sites when gRNA complementary nucleotides are truncated from 20 nt to 17 or 18 nt. Off-target mutations are screened out in the same predicated off-target sites, while at 9 out of 15 off-target sites, the respective mutation frequencies are not significantly different between tru-RGNs and std-RGNs. Moreover, off-target mutation frequency at one off-target site (OT3–2) is significantly increased by tru-gRNAs instead of std-gRNAs (22.0 *vs*. 3.7%). Similar results are observed in other previous studies in which increased off-target mutation frequencies resulted from tru-RGN use (tru-RGNs: 6.88% *vs.* std-RGNs: 3.88%) in U2OS.EGFP cells^13^.

Moreno-Mateos *et al.*^20^ found that the most efficient alternatives to canonical gRNAs are gRNAs shorter at the 5′ end by 1–2 nt or gRNAs of canonical length but with one mismatch in the 5′ GG region. The gene editing activity of alternative gRNAs has decreased according to GG sequence variants in order. We have designed truncated gRNAs with two or three nucleotides deleted, at Site Three where tru-gRNA is 17 nt, where target mutation efficiency on cells is significantly decreased, and there is no increase of mutation efficiency in newborn mice. This implies that truncation of gRNA to 17 nt might be considered the limit for gRNA activity. Off target mutations in *FVII* mutant mice are observed in 17 nt gRNA (OT3–2) at 29.3% (Supplementary Table S2). It was reported that shortening canonical gRNAs at 5′- by 4 nt to 16 nt tru-gRNA, has significantly reduced on targeting efficiency^13^. At Site One and Site Two where gRNAs are truncated to 18 nt, and GC ratio in gRNAs were 66.7% (tF7–1) and 61.1% (tF7–2), the on target mutation efficiencies of cells and animals are different. Compared with std-gRNA, tF7–1 gRNA has induced a decreased mutations in cultured cells, but has significantly increased the efficiency of generating mutant mice. On the other hand, tF7–2 has increased mutations in both cells and animals. This suggests that the editing capacity of tru-RGN is site-dependent. It is recommended that, prior to generating KO animals, gRNAs should be first tested in cell lines to screen out the best construct from several designed gRNAs. This approach can save time and reduce the use of animal resource in animal studies. Although we have taken precautions in designing gRNAs, it is often necessary to remove one or more nucleotides when the gRNA-encoding oligomer is cloned into an RGN expression plasmid at *Bsm*BI or *Bbs*I restriction sites^21^,^22^, or into a T7 promoter reading frame that requires a 5′-GG region for start of RNA *in vitro* transcription^23^,^24^. In our case, 4–5 nucleotides are added to create an artificial *Bbs*I site (Supplementary Table S3) for constructing recombinant gRNA expression vectors. In addition, 5′-GG transcription start region was generated with one or two G added (Supplementary Table S4). Thus, truncated gRNAs can induce different off-target mutations over different donor cells, target sites, and mismatches involved in complementary nucleotides^25^.

We found that both tru-RGNs and std-RGNs yield KO mice rarely carrying off-target mutations compared to transfected murine cells. Only one predicted off-target mutation (OT3–2, 29.3%) was found in tru-RGN-mutagenized mice, while off-target mutations were not observed in other mice. At the same OT3–2 site, the significant increase of off-target mutation was observed in cultured NIH/3T3 cells. These results are supported by other studies that reported lower frequencies of undesired Cas9-induced mutations in zygotes/embryos, when compared to embryonic stem cells^26^. Ma *et al.*^27^ also noted a lower off-target mutation frequencies using gRNA injection into rat zygotes. The difference in off-target mutation frequencies between cells and embryos could occur because higher levels of RGNs are persistently expressed in transfected cells, and edit genomic DNA intensely over a long period of time during cell culture, which provides more opportunity for RNA-DNA interaction. In order to reduce gRNAs and Cas9 nuclease expressed in cells, Fu *et al.*^2^ transfected less amount of gRNAs and Cas9 expression plasmids that decreased the mutation rate at on-target site, but did not appreciably change the relative rates of off-target mutations. However, during embryo microinjection, a lower concentrations of gRNAs and Cas9 mRNAs are co-injected into zygote, and therefore the specific on target mutations are decreased^23^. In our case, plasmid vector was transfected into cells, but only mRNAs were injected into embryos. This may be true that plasmid DNA can transcribe RNA longer in the cells, thus off-target mutations are higher than that by RNA injection in the embryos. It is reasonable to assume that off-target mutations are much more decreased^28^. Additional reason may be attributed to the induction of RGNs, of dominant lethal mutations at predicated and/or unknown off-targeting sites which are located in important genes critical for fetal development. These off-targeting mutations, in turn, result in embryo degeneration and/or fetal death during pregnancy. This implies that RGNs may represent a lower risk of off-target mutagenesis in KO mice compared to transfected cells.

Tru-RGNs can induce both biallelic and monoallelic mutations in *FVII* gene. We found that all biallelic *FVII* deficient mice (*FVII*^*−/−*^) died several days after birth, whereas mice with monoallelic mutations (*FVII*^+/−^) were alive, although some of them suffered of limb deformation. *FVII* gene can be inactivated by disrupting the entire coding sequence for the mature FVII protein^29^,^30^. Rosen *et al.*^30^ reported that FVII-deficient (*FVII*^*−/−*^) embryos developed normally, but 70% of the *FVII*^*−/−*^ newborn neonates suffered of fatal-abdominal bleeding, and the majority of remaining neonates died from intracranial hemorrhage before 24 days of age. At 9.5 day of embryonic development, FVII maternal–fetal transfer was undetectable. They suggested that the survival of embryos did not depend on cellular receptor tissue factor (TF)-FVII, responsible for initiating fibrin formation. Mice carrying *FVII*^+/−^ genotype have demonstrated substantial phenotypic changes such as prolonged PT in plasma. In our study, targeted sites are located in exon 2 of *FVII* gene, and any mutations occurring in these sites significantly affect their gene functions, such as completely inactivating FVII expression (Supplementary Table S1). The deduced amino acid sequences are shown as pre-mature termination of translation. Our results of prolonged PT and lowered (half) adult plasma FVII levels in heterozygous *FVII* KO mice (*FVII*^+/−^), strongly support the hypothesis that *FVII*^+/−^ mice only express half amount of FVII protein compared to wild type mice (*FVII*^*+/+*^), and that FVII is biallelicly expressed in the plasm of wild type (WT) mice. *FVII*-KO mouse model can be utilized in cardiovascular disease research and pharmaceutical developments.

In conclusion, truncated gRNAs together with Cas9 mRNAs (tru-RGNs) can be effectively used to generate *FVII* knockout mice with a much higher efficiency than using standard RGNs, although in a site-dependent manner. RGNs editing activity can be altered to a certain extent, depending upon where truncated gRNAs to 17/18 complementary nucleotides are located at different target sites. When the gene-edited KO mice are generated by RGNs microinjection into zygotes, the frequency of animals carrying off-target mutation is much lower than cells mediated by expressed RGNs and their cellular genome editing.

## Methods

### Animals

All protocols for animal treatment were approved by the Animal Care and Use Committees of Nanjing Normal University (NSD-2013–30). This study was also carried out in accordance with the recommendation in the Guided for the Care and Use of Laboratory Animals of the National Institutes of Health. Mice were maintained in an SPF animal facility at Nanjing Normal University and bred in IVC cages (4 mice per cage) with free access to food and water. Mice were kept under 12:12 h light: dark cycles in a room maintained at 24 ± 2 °C and 50 ± 20% relative humidity. A proper anesthesia before any procedures was performed to all animals which were part of the experiments.

### Construction of tru-RGN and std-RGN expression plasmids

Target sites at the second exon of the *FVII* gene (Supplementary Fig. S1) were analyzed by the MIT CRISPR Design Tool^22^. Off-target sites were identified in parallel, and gRNAs with the highest scores were chosen. Tru-gRNAs contained 17 or 18 target complementary nucleotides shortened from std-gRNAs. Oligomers encoding gRNAs were synthesized (Supplementary Table S4), annealed, and cloned into the PX459 vector (#48139, Addgene) at its *Bbs*I site as previously described^22^. An artificial *Bbs*I restriction site was generated for each vector by adding 4–5 nucleotides (Supplementary Table S3). Recombinant plasmids were sequenced and subsequently used for cell transfection.

### gRNA and Cas9 mRNA *in vitro* transcription

For the *in vitro* synthesis of gRNAs, the T7 promoter was inserted upstream of gRNAs by PCR using oligomers as previously described^31^, and 5′-GG transcription start region was arbitrarily created (Supplementary Table S4). The T7-gRNAs were gel-purified for *in vitro* transcription. Both std-gRNAs and tru-gRNAs were transcribed *in vitro* with the MEGAshortscript Kit (AM1354, Ambion). The pCAG-T3-hCAS-pA (#48625, Addgene) plasmid was digested by *Nru*I, and the DNA fragment encoding Cas9 was recovered by gel extraction for *in vitro* transcription. Cas9 mRNA was transcribed with the mMESSAGE mMACHINE^®^ T3 Transcription Kit (AM1348, Ambion). All RNAs were purified with the MEGAclear Transcription Clean-Up Kit (AM1908, Ambion) and stored at −80° C until use.

### Transfection of NIH/3T3 cells

Mouse embryonic fibroblast cells (NIH/3T3; ATCC, CRL-1658) were maintained in Dulbecco’s modified Eagle’s medium (DMEM, Life Technologies) supplemented with 10% fetal bovine serum (FBS, HyClone) at 37 °C with 5% CO_2_. Plasmid RGN DNA at the concentration of 50 ng/μL was used to transfect cells. Cells were transfected at 70–80% confluency using electroporation (ECM2001, BTX, USA) at 300 V with 1 μs and one pulse in a 0.2-cm cuvette with electro-transfection solution (272 mM sucrose, 7 mM K_2_HPO_4_, 1 mM MgCl_2_). Transfected cells were cultured in medium supplemented with 2 μg/ml puromycin after replating at 24 h and addition of fresh medium after 72 h. Cells that reached 80% confluency were recovered for genomic DNA extraction, 200 μl cell suspension containing 5 × 10^5^ cells was mixed with 400 μl Lysis Solution and incubate at 65 °C for 5 min. The transfected cells were screened 72 h by puromycin and cultured 2 d for colony counting. Colony efficiency = No. colonies/total of transfected cells × 100%. DNA extraction was carried out using Genomic DNA Purification KIT (K0512, Thermo) according to the manufacturer’s instructions.

### gRNA and Cas9 mRNA co-injection into murine zygotes and breeding

Female mice (C57BL/6 J, 4–6 weeks old) were used as embryo donors for superovulation. After mating, resultant fertilized embryos were collected from the oviducts and cultured in KSOM medium as previously described^32^,^33^. Microinjection was performed using an Olympus IX71 inverted microscope with the Narishige microinjection system. gRNA (50 ng/μL) and Cas9 mRNA (100 ng/μL) were mixed and injected into the cytoplasm of the zygotes with visible pronuclei in M2 medium as previously described^23^. The injected embryos were cultured overnight in KSOM medium at 37 °C with 5% CO_2_. Embryos at the two-cell stage were transferred into oviducts of pseudopregnant ICR females. Tails of 3-week-old live pups were collected for extraction of genomic DNA. Approximately 1 cm long of tail from each mice was digested with Proteinase K butter. DNA was extracted using Phenol/Chloroform and ethanol as previously described^34^. Presumptive heterozygous *FVII* founders (*FVII*^+/−^) were mated with wild-type mice to determine whether offspring followed the Mendelian hereditary pattern.

### PCR amplification of on-target and off-target sites

Transfected cell and mouse tail genomic DNA were used as template with high fidelity DNA polymerase (New England BioLabs, NEB). Target or off-target regions (500–700 bp) were amplified by PCR primers (Supplementary Table S5). Most sites were amplified successfully (95 °C, 20 s; 58 °C, 20 s; 72 °C, 40 s) after 32 cycles. PCR products were analyzed by agarose gel electrophoresis to verify amplicon size and quality.

### T7EI assays for determining mutation frequencies

T7EI assays were performed as previously described^31^. Briefly, 500 ng purified PCR products from transfected cell DNA were denatured and annealed to form heteroduplexes by incubating at 95 °C for 10 min and cooling to 25 °C at 5 °C /min. For mouse tail genomic DNA, 250 ng PCR products were mixed with 250 ng wild-type (WT) genomic DNA PCR products and then denatured and annealed. Hybridized PCR products were digested with T7 Endonuclease I (T7EI, M0302L, NEB) for 30 min at 37 °C. Reaction products were analyzed by agarose gel electrophoresis. On/off target mutation frequencies were calculated using the following formula as previously described^22^: 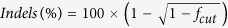. *f*_*cut*_ = amount of digested PCR fragments compared to total PCR fragments. Further confirmation of mutations was performed by either PCR sequencing or TA cloning-sequencing described below.

### Cloning and Sanger sequencing to identify sequence modifications

Target region amplicons were cloned into a TA-cloning vector (PCR Cloning Kit, E1202S, NEB) and transformed into *E. coli* DH5α competent cells as previously described^22^. Briefly, 50 ng PCR product was added into 100 μl of ice-cold chemically competent DH5α cells. The cell mixtures were incubated on ice for 10 min, heat-shocked it at 42 °C for 30 s and returned it immediately to ice for 2 min, finally plated onto on LB plate containing 100 μg/ml ampicillin and incubated at 37 °C overnight. Plasmid DNA was isolated and sequenced by commercial sequencing company (Sangon Biotech).

### Thrombin reaction in mice

Blood from WT mice and putatively KO mice was collected by orbital venipuncture into plastic tubes containing 1/10 volume 0.129 M buffered trisodium citrate. Plasma was recovered by centrifugation at 4 °C, 2500 × *g* for 15 min, transferred into plastic tubes, and stored at 4 °C for immediate use or at −80 °C for later use. Coagulant activity of plasma FVII was measured by a one-stage prothrombin time (PT)-based assay^35^. Fifty microliters blood plasma were incubated at 37 °C for 3 min and immediately mixed with 100 μl pre-warmed thromboplastin reagent (Prothrombin Time Assay Kit, F007, Nanjing Jiancheng BIO). The PT was then recorded.

### Western blot analysis of FVII expression in mice

Total plasma proteins were quantified with the BCA assay kit (GK5011, Shanghai Generay Biotech). Thirty micrograms total plasma proteins were separated by SDS-PAGE and transferred to a PVDF membrane. Immunoblotting was carried out with FVII-specific antibody (1:1000 rabbit anti-FVII antibody, Lannuo Biotechnologies Wuxi Inc.). Primary antibodies were visualized with goat anti-rabbit IgG-HRP secondary antibody (SC-2004, Santa Cruz Biotechnology) using an Enhanced ECL Chemiluminescence Detection Kit (E411–04, Vayme).

### Statistical analyses

The data on genomic editing and off-target mutation frequencies induced in cells and newborn mice by RGNs, were analyzed using the SPSS software (SPSS 18.0, IBM). Percentage data in each replicate were arc-sine transformed and subjected to one-way ANOVA. Means were compared by Fisher’s least significant difference test (PLAS). Statistical significance was defined as *P* < 0.05.

## Additional Information

**How to cite this article**: An, L. *et al.* Efficient generation of FVII gene knockout mice using CRISPR/Cas9 nuclease and truncated guided RNAs. *Sci. Rep.* **6**, 25199; doi: 10.1038/srep25199 (2016).

## Supplementary Material

Supplementary Information

## Figures and Tables

**Table 1:** Mutation frequencies of the *FVII* gene in transfected cells induced by tru-RGNs and std-RGNs.

ID gRNAs	Target sites (5′-3′)*	Length(nt)	Sites inexon 2	Mutation frequency(%) Mean ± SEM
F7–1	AAGGCGTGCCAACTCACTCC TGG	20	43–63	23.6 ± 0.5^a^
tF7–1	GGCGTGCCAACTCACTCC TGG	18	45–63	12.1 ± 0.2^b^
F7–2	GCGTGCCAACTCACTCCTGG AGG	20	46–66	30.1 ± 0.9^a^
tF7–2	GTGCCAACTCACTCCTGG AGG	18	48–66	49.5 ± 1.0^b^
F7–3	GGAGCTTTGGCCCGGCTCTC TGG	20	67–87	10.9 ± 1.3
tF7–3	GCTTTGGCCCGGCTCTC TGG	17	70–87	7.7 ± 0.9^b^

^a,b^ values with the different numbers within the same column showed significant differences (*P* < 0.05). The statistical comparison was performed in pair between std-RGNs and tru-RGNs, that was F7–1 vs. tF7–1, F7–2 vs. tF7–2, F7–3 vs. tF7–3 (n = 3). *Either TGG or AGG at 3′-end of each gRNAs was shown as protospacer adjacent motif (PAM) necessary for gRNA recognition. The target sites were counted from the first nucleotide of exon 2 in *FVII* (GenBank Accession No. U66079, as shown in Fig. 1S A).

**Table 2:** Generating *FVII* KO mice with gRNA and Cas9 mRNA co-injection.

IDgRNAs	No. injectedembryos	No. embryostransferred	No. ofReplicates	No.recipients	No.newborns	No. of newborns containing mutant allele(s)*			Mutant mice / total mice tested (%) (Mean ± SEM)
						Total No.mutantmice	No. with oneallele (Alive)	No. with twoalleles (Deceased)	
F7–1	78	71	3	3	18	1	1 (1)	0	3.7 ± 3.7^a^
tF7–1	98	98	3	3	38	19	1 (1)	18 (18)	55.0 ± 5.0^b^
F7–2	110	106	3	4	24	8	2 (2)	6 (6)	35.8 ± 5.8^a^
tF7–2	75	67	3	3	20	15	15 (15)	0	80.1 ± 7.4^b^
F7–3	80	77	3	3	6	2	2 (2)	0	27.8 ± 14.7^a^
tF7–3	120	115	3	4	20	8	8 (8)	0	39.4 ± 6.1^a^

^a,b^ values with the different numbers within the same column showed significant differences (*P* < 0.05). The experiment was performed with three replicates. The statistical comparison was performed in pair between std-RGNs and tru-RGNs that was F7–1 vs. tF7–1, F7–2 vs. tF7–2, F7–3 vs. tF7–3. *all newborns with one mutant allele survived, while those with double mutant alleles were survived, but deceased shortly after birth.

**Table 3:** Comparison of off target mutation in both mouse cell lines and mice induced by tru-RGNs and std-RGNs.

Target sites	Recognition sites		Mutation frequencies in cells (%)		Mutant mice / total mice tested (%)	
	std-RGNs (20 nt)	Tru-RGNs (17/18 nt)	Std-gRNA Mean ± SEM	Tru-gRNA Mean ± SEM	Std-gRNA Mean ± SEM	Tru-gRNA Mean ± SEM
*FVII* site 1 (on-target)	AAGGCGTGCCAACTCACTCC TGG	GGCGTGCCAACTCACTCC TGG				
OT1–1	AGGATGGGCCAACTCACTCC TGG	GATGGGCCAACTCACTCC TGG	13.3 ± 0.8^a^	13.1 ± 0.3^a^	0^a^	0^a^
OT1–2	AGGATGGGCCAACTCACTCC TGG	GATGGGCCAACTCACTCC TGG	6.6 ± 0.2^a^	6.2 ± 0.3^a^	0^a^	0^a^
OT1–3	AAGCCCTCCTAACTCACTCC CAG	GCCCTCCTAACTCACTCC CAG	0^a^	0^a^	0^a^	0^a^
OT1–4	AAGCCATCCAAACTCACTCC TGG	GCCATCCAAACTCACTCC TGG	21.4 ± 0.6^a^	20.3 ± 1.0^a^	0^a^	0^a^
OT1–5	ACTGTGTGCCACCTCACTCC TGG	TGTGTGCCACCTCACTCC TGG	2.1 ± 0.4^a^	0^b^	0^a^	0^a^
*FVII* site 2 (on-target)	GCGTGCCAACTCACTCCTGG AGG	GTGCCAACTCACTCCTGG AGG				
OT2–1	GCTGGCAAACTCACTCCTGG AAG	TGGCAAACTCACTCCTGG AAG	4.4 ± 0.4^a^	3.0 ± 0.1^b^	0^a^	0^a^
OT2–2	GGCTGCCACCTCACTCCTGG AGG	CTGCCACCTCACTCCTGG AGG	0^a^	0^a^	0^a^	0^a^
OT2–3	GTGTGTCTACTCACTCCTGG GGG	GTGTCTACTCACTCCTGG GGG	0^a^	0^a^	0^a^	0^a^
OT2–4	CTGTCCCAATTCACTCCTGG TAG	GTCCCAATTCACTCCTGG TAG	0^a^	0^a^	0^a^	0^a^
OT2–5	AGGTACCAGCTCACTCCTGG GGG	GTACCAGCTCACTCCTGG GGG	8.5 ± 0.5^a^	6.0 ± 0.3^b^	0^a^	0^a^
*FVII* site 3 (on-target)	GGAGCTTTGGCCCGGCTCTC TGG	GCTTTGGCCCGGCTCTC TGG				
OT3–1	GGGGCTTTGGCCAGGCTCTC AGG	GCTTTGGCCAGGCTCTC AGG	0^a^	0^a^	0^a^	0^a^
OT3–2	AGGGCCTTGGCCCGGCTCTC GGG	GCCTTGGCCCGGCTCTC GGG	3.7 ± 0.5^a^	22.0 ± 0.6^b^	0^a^	29.3 ± 7.1^b^
OT3–3	TAAACTTTGACCCGGCTCTC TAG	ACTTTGACCCGGCTCTC TAG	5.7 ± 0.4^a^	5.2 ± 0.4^a^	0^a^	0^a^
OT3–4	ACAGCTTTGGCCAGGCTCTC GAG	GCTTTGGCCAGGCTCTC GAG	6.2 ± 0.3^a^	3.2 ± 0.2^b^	0^a^	0^a^
OT3–5	ACAGCTTTGGCCAGGCTCTC GAG	GCTTTGGCCAGGCTCTC GAG	7.2 ± 0.1^a^	5.6 ± 0.2	0^a^	0^a^

^a,b^ values with the different numbers within the same row showed significant differences (*P* < 0.05). The frequencies of mutation at predicated off-target (OT) sites were compared separately in either cell lines or newborn mice between each tru-gRNA and std-gRNA group. At each OT site, the different nucleotides mismatch to the targeted sequence were marked in grey highlighted. The mutations were detected by T7E1 assay together with PCR-sequencing or TA cloning-sequencing.
