# Supplementary material for: Efficient generation of FVII gene knockout mice using CRISPR/Cas9 nuclease and truncated guided RNAs
Source: Sci Rep. 2016 May 3;6:25199. doi: 10.1038/srep25199 (PMC4853708; doi:10.1038/srep25199)
Supplement: Supplementary Information [file srep25199-s1.doc]

**Supplementary Information**

**Efficient generation of *FVII* gene knockout mice using CRISPR/Cas9 nuclease and truncated guided RNAs**

Liyou An^a^, Yeshu Hu^a^, Shiwei Chang^a^, Xiumei Zhu^a^, Pingping Ling^a^, Fenli Zhang^a^, Jiao Liu^a^, Yanhong Liu^a^, Yexiang Chen^a^, Lan Yang^b^, Giorgio Antonio Presicce^c^ and Fuliang Du^a,d^*****

# ^a^Jiangsu Key Laboratory for Molecular and Medical Biotechnology, College of Life Sciences, Nanjing Normal University, Nanjing 210046, P R China

# ^b^Lannuo Biotechnologies Wuxi Inc., Wuxi 214000, P R China

# ^c^ARSIAL, Rome, Italy

# ^d^Renova Life, Inc., College Park, Maryland 20742, USA

# *Corresponding author:

# Fuliang Du, PhD

# Professor

# Jiangsu Key Laboratory for Molecular and Medical Biotechnology

# College of Life Sciences, Nanjing Normal University

# #1 Wenyuan Rd, Nanjing 210046, P R China

# Tel: +86-25-85898011

# Email: fuliangd@njnu.edu.cn


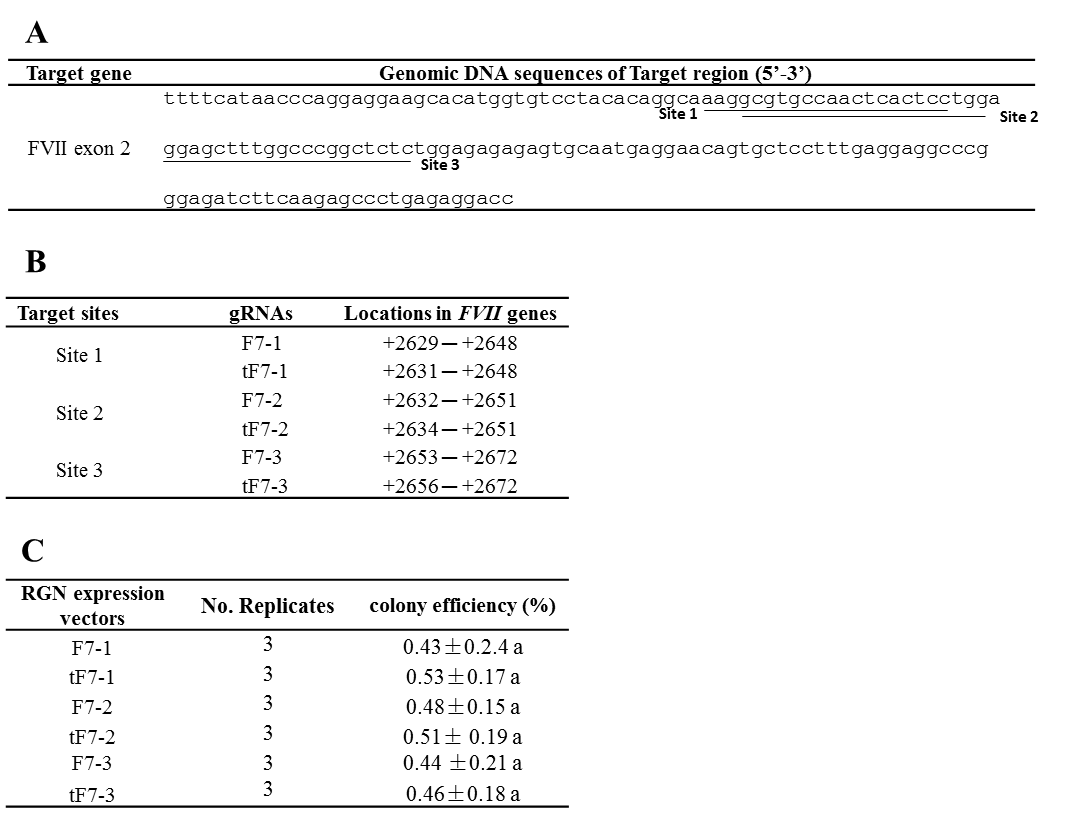


**Figure S1.** (A) The gRNAs recognizing sequences were labeled by lines below the whole exon 2 cDNA sequences (GenBank Accession No. U66079). (B) Recognition region of Cas9 nuclease guided by gRNAs were listed as its location in *FVII* genomic DNA sequences (GenBank Accession No. U66079). (C) The transfected cells were screened 72 h by puromycin and cultured 2 d for colony counting. Colony efficiency = No. colonies / total of transfected cells ×100%. a values in the column showed no significant differences (*P*>0.05).

**Table S1. Deduced amino acid sequence of mutant FVII proteins in KO mice**

| **Mice** | **Amino acid sequences** | **Residues** |
| --- | --- | --- |
| FVII.pro MVPQAHGLLLLCFLLQLQGPLGTAVFITQEEAHGVLHRQRRANSLLEELWPGSLERECNEEQCSFEEAREIFKSPERTKQFWIVYSDGDQCASNPCQNGG 100  H68.pro MVPQAHGLLLLCFLLQLQGPLGTAVFITQEEAHGVLHRQRRANSRSFGPALWRESAMRNSAPLRRPGRSSRALRGPSSSGLFTVMGTSVPRIHVRTEVPA 100  H69.pro MVPQAHGLLLLCFLLQLQGPLGTAVFITQEEAHGVLHRQRRANFGPALWRHSAMRNRAPLKTSGRSSRALRGPSSSGLFTVMGTSVPRIHVRTEVPARII 100  H78.pro MVPQAHGLLLLCFLLQLQGPLGTAVFITQEEAHGVLHRQRRANSLGERVQ 50  O68.pro MVPQAHGLLLLCFLLQLQGPLGTAVFITQEEAHGVLHRQRRANSEELGPALWRESAMRNSAPLRRPGRSSRALRGPSSSGLFTVMGTSVPRIHVRTEVPA 100  FVII.pro TCQDHLKSYVCFCLLDFEGRNCEKSKNEQLICANENGDCDQYCRDHVGTKRTCSCHEDYTLQPDEVSCKPKVEYPCGRIPVVEKRNSSSRQGRIVGGNVC 200  H68.pro RIISSLTSASAS 112  H69.pro SSLTSASAS 109  O68.pro RIISSLTSASAS 112  FVII.pro PKGECPWQAVLKINGLLLCGAVLLDARWIVTAAHCFDNIRYWGNITVVMGEHDFSEKDGDEQVRRVTQVIMPDKYIRGKINHDIALLRLHRPVTFTDYVV 300  FVII.pro PLCLPEKSFSENTLARIRFSRVSGWGQLLDRGATALELMSIEVPRLMTQDCLEHAKHSSNTPKITENMFCAGYMDGTKDACKGDSGGPHATHYHGTWYLT 400  FVII.pro GVVSWGEGCAAIGHIGVYTRVSQYIDWLVRHMDSKLQVGVFRLPLL 446 | | |

The amino acid sequences of mutant FVII protein were deduced from heterozygous H68, H69, H78 and O68 mouse (*FVII*^+/-^). The deduced peptides were all shifted at the positions of mutation (labeled in grey highlighted), while compared with wild type (WT) FVII amino acid sequence. These deduced sequences after mutations were completely different and not matched with the sequence of WT FVII; in addition, these deduced amino acid sequences were all pre-maturely terminated.

**Table S2. DNA sequences of off-target** mutations induced by RGNs in mice

| **Founder** | | **Target sequences (5’-3’)** | **Indels** |
| --- | --- | --- | --- |
| RGNs F7-3 induced mutations in OT3-2 site | | |  |
| WT | TGGCGGAAGAGGGCCTTGGCCCGGCTCTCGGGAAGTGGCCCTCCGTTCAGCACACAGTCA | |  |
| H60 | TGGCGGAAGAGGGCCTTGG-----CTCTCGGGAAGTGGCCCTCCGTTCAGCACACAGTCA | | ∆5 nt |
| H61 | TGGCGGAAGAGGGCCTTGG-------------AAGTGGCCCTCCGTTCAGCACACAGTCA | | ∆13 nt |
| H62 | TGGCGGAAGAGGGCCTTGGCC--------------TGGCCCTCCGTTCAGCACACAGTCA | | ∆14 nt |
| H63 | TGGCGGAAGAGGGCCTTGG------TCTCGGGAAGTGGCCCTCCGTTCAGCACACAGTCA | | ∆6 nt |
| H64 | TGGCGGAAGAGGGCCT-------------------------------CAGCACACAGTCA | | ∆31 nt |
| H85 | TGGCGGAAGAGGGCCT---------------GAAGTGGCCCTCCGTTCAGCACACAGTCA | | ∆15 nt |

The target sequences were labeled as underlines in wildtype. Mutations of nucleotide (nt) deletions were shown as “-”. They were off-target mutations at OT3-2 (Chr4:129166779) induced by tF7-3 in mice. The mutations were detected by T7E1 assay together with PCR-sequencing or TA cloning-sequencing.

**Table S3**. DNA sequences for constructing recombinant gRNA expression vectors

| **gRNA name** | **Target site (5’-3’)** | **Length (bp)** | | **Synthesized DNA sequence** | |
| --- | --- | --- | --- | --- | --- |
|  |  |  |  | **Sense (5’-3’)** | **Antisense (5’-3’)** |
| **std-gRNAs** |  |  | |  |  |
| F7-1 (site 1) | AAGGCGTGCCAACTCACTCC | | 20 | caccgAAGGCGTGCCAACTCACTCC | aaacGGAGTGAGTTGGCACGCCTTc |
| F7-2 (site 2) | GCGTGCCAACTCACTCCTGG | | 20 | caccGCGTGCCAACTCACTCCTGG | aaacCCAGGAGTGAGTTGGCACGC |
| F7-3 (site 3) | GGAGCTTTGGCCCGGCTCTC | | 20 | caccGGAGCTTTGGCCCGGCTCTC | aaacGAGAGCCGGGCCAAAGCTCC |
| **tru-gRNAs** |  | |  |  |  |
| tF7-1 (site 1) | GGCGTGCCAACTCACTCC | | 18 | caccGGCGTGCCAACTCACTCC | aaacGGAGTGAGTTGGCACGCC |
| tF7-2 (site 2) | GTGCCAACTCACTCCTGG | | 18 | caccGTGCCAACTCACTCCTGG | aaacCCAGGAGTGAGTTGGCAC |
| tF7-3 (site 3) | GCTTTGGCCCGGCTCTC | | 17 | caccGCTTTGGCCCGGCTCTC | aaacGAGAGCCGGGCCAAAGC |

A *Bbs*I restriction site was artificially generated by adding “caccg” (F7-1) or “cacc” (F7-2, F7-3, tF7-1, tF7-2, tF7-3) (underlined lower cases) in synthesized DNAs at 5’-end of sense gRNAs (Sense, 5’-3’), and by adding “aaac” (underlined lower cases) at 5’-end of antisense gRNAs (Antisense, 5’-3’). These sequences were used to clone them into *PX459* RNA expression vector.

**Table S4. Oligomers used as templates for *in vitro*** transcription of gRNAs

| **gRNA** | | **Synthesized oligomer** |
| --- | --- | --- |
| **Forward sequences for std-gRNAs (5’-3’)** | | |
| F7-1 | GATAATACGACTCACTATAGGAAGGCGTGCCAACTCACTCCGTTTTAGAGCTAGAAATA | |
| F7-2 | GATAATACGACTCACTATAGGCGTGCCAACTCACTCCTGGGTTTTAGAGCTAGAAATA | |
| F7-3 | GATAATACGACTCACTATAGGAGCTTTGGCCCGGCTCTCGTTTTAGAGCTAGAAATA | |
| **Forward sequences for tru-gRNAs (5’-3’)** | | |
| tF7-1 | GATAATACGACTCACTATAGGCGTGCCAACTCACTCCGTTTTAGAGCTAGAAATA | |
| tF7-2 | GATAATACGACTCACTATAGGTGCCAACTCACTCCTGGGTTTTAGAGCTAGAAATA | |
| tF7-3 | GATAATACGACTCACTATAGGCTTTGGCCCGGCTCTCGTTTTAGAGCTAGAAATA | |
| **Reverse sequence for all gRNAs (5’-3’)** | | |
| CTGCAGCACCGACTCGGTGCCACTTTTTCAAGTTGATAACGGACTAGCCTTATTTTAACTTGCTATTTCTAGCTCTAAAAC | | |

T7 promotor was grey highlighted, and two protective nucleotides (GA) were added in front of the promoter. The sequences of gRNAs were underlined. Templets of gRNA transcription were amplified by PCR using forward and reverse oligomers.

**Table S5. Primers for PCR amplification** of on-target and off-target products

| **Primers** | **Sequences (5’-3’)** | **gRNAs recognition site *** | |
| --- | --- | --- | --- |
| Primers for detecting on-target mutations in *FVII* gene by PCR amplification | | | |
| F7-667-f1 | GCACCTTCCGTTCCTTGAG | | Chr8: + 13028687 |
| F7-667-r1 | CAGCCAGTGTAGTTTATGAGTTGTA | |
| Primers for detecting off-target mutations induced by RGNs of tF7-1 and F7-1 | | | |
| C7-#1-OT1f | ATAGCTCCATAAGTCAAAG | | OT1-1, Chr8:+101856136 |
| C7-#1-OT1r | ATTCTGCACTGGCATC |  |
| C7-#1-OT2f | GAGCCAAATTGAACGC |  | OT1-2, Chr16:+59710578 |
| C7-#1-OT2r | GAGCACAAAGCCGATG |  |
| C7-#1-OT3f | GGAAACAGAGCAGGAAGTG | | OT1-3, Chr7:-80997272 |
| C7-#1-OT3r | CCTAGCAAAGCAGGACGT | |
| C7-#1-OT4f | CTGAGAAACTGCTGGGTAA | | OT1-4, Chr14:-12989091 |
| C7-#1-OT4r | AAAGTTAATGGCACAAGTCA | |
| C7-#1-OT5f | ATCGCAGTTCTTCACAATCC | | OT1-5, Chr9:+62678536 |
| C7-#1-OT5r | TCACCATCCTCCTGCCTC |  |
| Primers for detecting off-target mutations induced by RGNs of tF7-2 and F7-2 | | | |
| C7-#2-OT1f | TGACTATTTTCTGCCATT |  | OT2-1, Chr3:+74794888 |
| C7-#2-OT1r | CTTAAACCACAACTGAGC |  |
| C7-#2-OT2f | ACACGAATACACGATGCA | | OT2-2, Chr8:+90339695 |
| C7-#2-OT2r | GGAAAGGAAACGGGAG |  |
| C7-#2-OT3f | GACACCATCCCTCCATC |  | OT2-3, Chr8:+12608082 |
| C7-#2-OT3r | ACTAGCCATTTCCCTCTG |  |
| C7-#2-OT4f | AGCCTAAGATACAGTTAGACCC | | OT2-4, Chr6:+114177350 |
| C7-#2-OT4r | GCTTTGAAGTCAGCAGGAG | |
| C7-#2-OT5f | CCATAGTACCCAGGATAGAACAG | | OT2-5, Chr2:+168307775 |
| C7-#2-OT5r | ACCTCACCTCAAGGGACAAC | |
| Primers for detecting off-target mutations induced by RGNs of tF7-3 and F7-3 | | | |
| C7-#3-OT1f | TTTACTACCTCTGCCTTGAA | | OT3-1, Chr2:-124900744 |
| C7-#3-OT1r | CTGTGCCTGCACCTACAT |  |
| C7-#3-OT2f | CAAAGCCAAAGTCGGTCAG | | OT3-2, Chr4:+129166779 |
| C7-#3-OT2r | GTCAGGGAGTCAAGAAAGAACA | |
| C7-#3-OT3f | GGGACATGCAGTGATTTAA | | OT3-3, Chr14:-62139520 |
| C7-#3-OT3r | TACCTCCTCTAGTGGTTGGA | |
| C7-#3-OT4f | CTGCTCTTTGGGTTCTTGG | | OT3-4, Chr14:-31117160 |
| C7-#3-OT4r | CCCTCTGTGGGTTGTCATT | |
| C7-#3-OT5f | CACTTGGGTTGAAATAGA | | OT3-5, Chr19:+22207269 |
| C7-#3-OT5r | CAAACCTAAAAGGGTATG | |

Forward primers (f) were designed at 250- 350 bp up-stream of gRNA recognition site. Reverse primers (r) were located at 250-350 bp down-stream of gRNA recognition site. Five sites with the highest potentials of off-target (OT) for each of gRNAs were amplified. Therefore, the total number of OTs corresponding three gRNA on-target sites was 15. * All sequences of gRNA recognition sites were searched from GeneBank (http://www.ncbi.nlm.nih.gov/genbank/).
